# Annexin A2 (ANXA2) regulates the transcription and alternative splicing of inflammatory genes in renal tubular epithelial cells

**DOI:** 10.1186/s12864-022-08748-6

**Published:** 2022-07-29

**Authors:** Jing Chen, Yuwei Liu, Shang Xia, Xujun Ye, Ling Chen

**Affiliations:** Department of Internal Medicine and Geriatrics, Zhongnan Hospital of Wuhan University, Wuhan University, NO.169 Donghu Road, Wuhan, 430071 Hubei China

**Keywords:** Annexin A2, Differentially expressed genes, Alternative splicing, Inflammation, Renal tubular epithelial cell

## Abstract

**Background:**

Renal inflammation plays a crucial role during the progression of Chronic kidney disease (CKD), but there is limited research on hub genes involved in renal inflammation. Here, we aimed to explore the effects of Annexin A2 (ANXA2), a potential inflammatory regulator, on gene expression in human proximal tubular epithelial (HK2) cells. RNA-sequencing and bioinformatics analysis were performed on ANXA2-knockdown versus control HK2 cells to reveal the differentially expressed genes (DEGs) and regulated alternative splicing events (RASEs). Then the DEGs and RASEs were validated by qRT-PCR.

**Results:**

A total of 220 upregulated and 171 downregulated genes related to ANXA2 knockdown were identified. Genes enriched in inflammatory response pathways, such as interferon-mediated signaling, cytokine-mediated signaling, and nuclear factor κB signaling, were under global transcriptional and alternative splicing regulation by ANXA2 knockdown. qRT-PCR confirmed ANXA2-regulated transcription of chemokine gene *CCL5*, as well as interferon-regulating genes *ISG15*, *IFI6*, *IFI44*, *IFITM1*, and *IRF7*, in addition to alternative splicing of inflammatory genes *UBA52*, *RBCK1*, and *LITAF*.

**Conclusions:**

The present study indicated that ANXA2 plays a role in inflammatory response in HK2 cells that may be mediated via the regulation of transcription and alternative splicing of inflammation-related genes.

**Supplementary Information:**

The online version contains supplementary material available at 10.1186/s12864-022-08748-6.

## Background

Chronic kidney disease (CKD) remains a major public health problem with limited available treatments [1]. A growing number of researches suggest that inflammation plays an important role in CKD [2, 3]. Inflammation in CKD is a complex network of interactions between kidney inherent cells and immune cells, accompanied by the recruitment of circulating monocytes, lymphocytes, and neutrophils [3]. Inflammatory pathways and signaling molecules, such as nuclear factor κB (NF-κB), monocyte chemoattractant protein-1, transforming growth factor β, interferon-γ (IFN-γ), tumor necrosis factor-α (TNF-α), and platelet-derived growth factor were found activated during CKD [4–7]. While several anti-inflammatory factors were found attenuated in CKD, such as interleukin (IL)-10 and bone morphogenetic protein-7 [7–9]. Restoration of the balance between pro- and anti-inflammatory signaling pathways is considered as a potential approach to treat CKD [7–9].

In this regard, the exploration of key upstream modulators in the kidney inflammation network has taken center stage. Annexin A2 (ANXA2), a 36-kD protein belongs to the annexin family, has been connected to a vast array of cellular functions, such as endocytosis, exocytosis, membrane domain organization, and translational regulation through RNA binding [10, 11]. Recent studies have proposed that ANXA2 plays a role in inflammation [12]. By modulating immunosuppressive responses, ANXA2 has been implicated in the process of tumor metastasis [13]. Furthermore, NLRP3 inflammasome activation was found elevated in dendritic cells of ANXA2-KO mice [14], as well as release of pro-inflammatory cytokines and superoxide [15, 16]. These revealed the anti-inflammatory role of ANXA2. However, the opposite function of ANXA2 in inflammatory reactions has also been reported. In rheumatoid arthritis, ANXA2 was believed to act as a pro-inflammatory actor by inducing expression of inflammatory cytokines TNF-α, IL-1β, and IL-6 [17]. Upregulation of ANXA2 had also been reported to accelerate fibroblast proliferation and fibrosis in several immune-mediated diseases [18]. The different roles of ANXA2 in inflammatory response, anti-inflammation, and pro-inflammation may depend on microcirculation. These findings revealed a strong correlation between ANXA2 and inflammatory system. However, there is a scarcity of research focusing on ANXA2 and kidney-related inflammation.

Besides regulating mRNA transcription, ANXA2 was also reported to have multiple roles in post-transcriptional regulation of gene expression [19, 20]. But the specific mechanisms remain to be studied. The significance of alternative splicing in post-transcriptional regulation has been found in many types of RNA-binding proteins, as the development of high-throughput sequencing technology [21] . Aa a RNA-binding protein with a wide range of interactions [11], ANXA2 might have potential roles in splicing regulation, which deserves to be deeply investigated. In the present study, we aim to explore whether ANXA2 had a modulatory effect on kidney inflammation. Through RNA-sequencing (RNA-seq) analysis, it would reveal whether ANXA2 regulates the expression and alternative splicing of key inflammatory response genes in renal tubular epithelial cells.

## Results

### RNA-seq data summary and exploration of DEGs

**Fig. 1:**
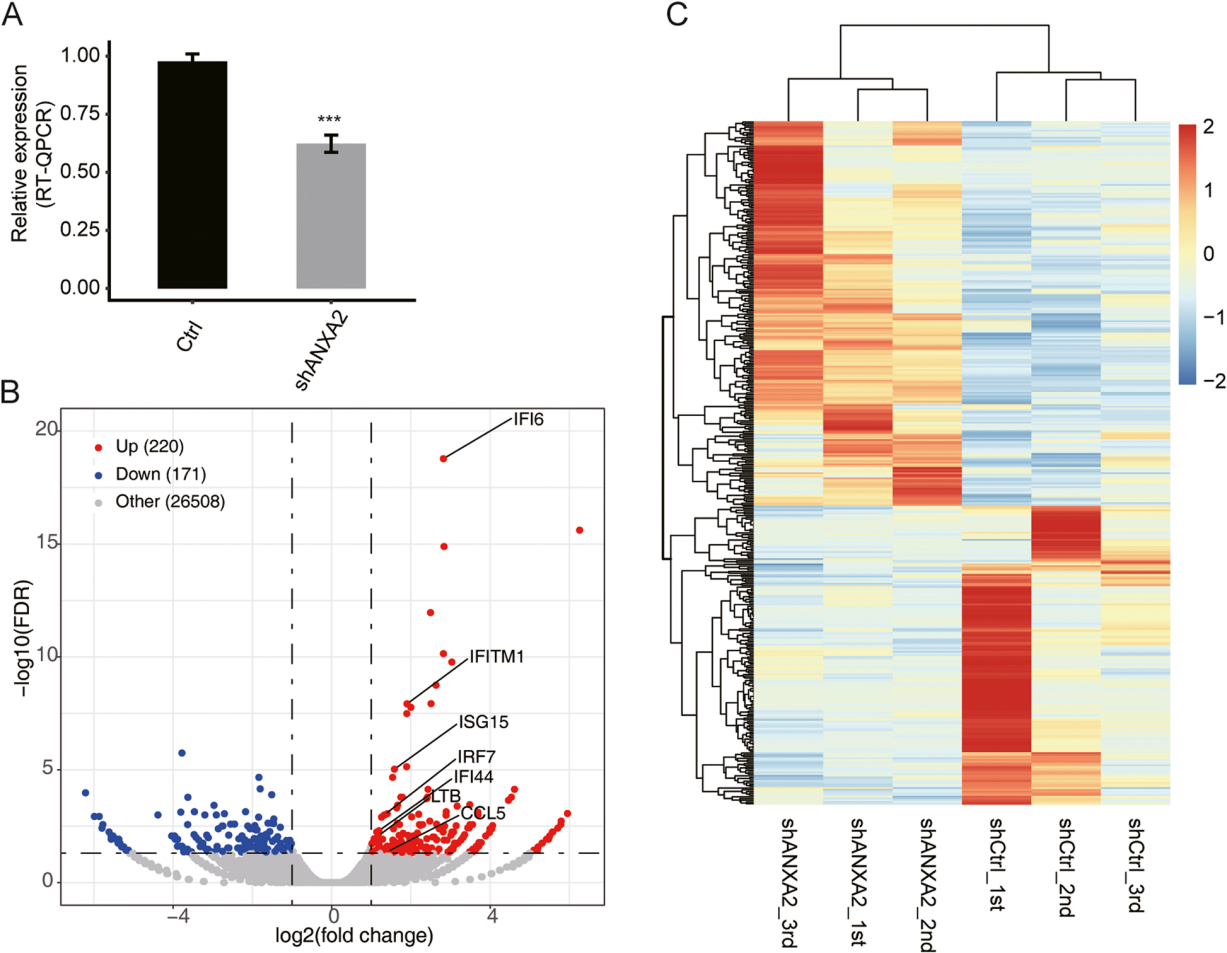
Differentially expressed genes (DEGs) in shANXA2 and shCtrl HK2 cells. **A** The level of ANXA2 mRNA expression by qRT-PCR in shCtrl vs shANXA2 cells. (*n* = 3, ***P < 0.001, calculated using the student’s t-test). **B** Volcano plots indicate gene expression in shCtrl vs shANXA2 cells. Genes with significant differential expression are shown in red (up-regulated) or blue (down-regulated), and genes that were not significantly differentially expressed are shown in grey. Filtering criteria (log2 fold change >1 or <-1 and FDR < 0.05) was set as the threshold to determine the DEGs. **C** Heatmaps for DEGs of shCtrl vs shANXA2 cells

To explore ANXA2-mediated transcriptional regulation, ANXA2 expression was knocked down using shRNA in HK2 cells. RNA-seq experiments were performed in ANXA2 knockdown (shANXA2, *n* = 3) and control samples (shCtrl, *n* = 3). As shown in Fig. 1A, the efficacy of ANXA2 knockdown was assessed by qRT-PCR. ANXA2 knockdown and control cells were used to construct cDNA libraries for sequencing on an Illumina HiSeq X Ten platform. The sequencing data were reviewed to ensure their reliability (shown in Supplementary Material Table 1). A total of 76.8 ± 7.0 M raw reads per sample were generated and 75.2 ± 8.0 M clean reads per sample were retained by the removal of adaptors and contaminating sequences. Among these, 70.2 ± 7.5 M were paired-end reads per sample, and an average of 67.8 ± 7.4 M read pairs per sample were aligned to the human genome. Gene and transcript quantifications were reassessed using Cufflinks [22], to compare gene expression patterns between individuals. FPKM values were then calculated. Using criteria of log2 fold change (>1 or <-1) and false discovery rate (FDR) of < 0.05 with the edgeR package, 220 upregulated and 171 downregulated genes related to ANXA2 knockdown were identified. A volcano plot was constructed to display the DEGs that were significantly associated with ANXA2 knockdown (shown in Fig. 1B). We found the DEGs that showed consistent expression patterns in three replicates by plotting a hierarchical clustering heatmap, and the shANXA2 samples were clearly separated from shCtrl samples (shown in Fig. 1C). The datasets generated and/or analysed during the current study are available in the GEO database, submission number GSE159360 (https://www.ncbi.nlm.nih.gov/geo/).

### ANXA2 regulates the expression of genes enriched in inflammatory response in HK2 cells

**Fig. 2:**
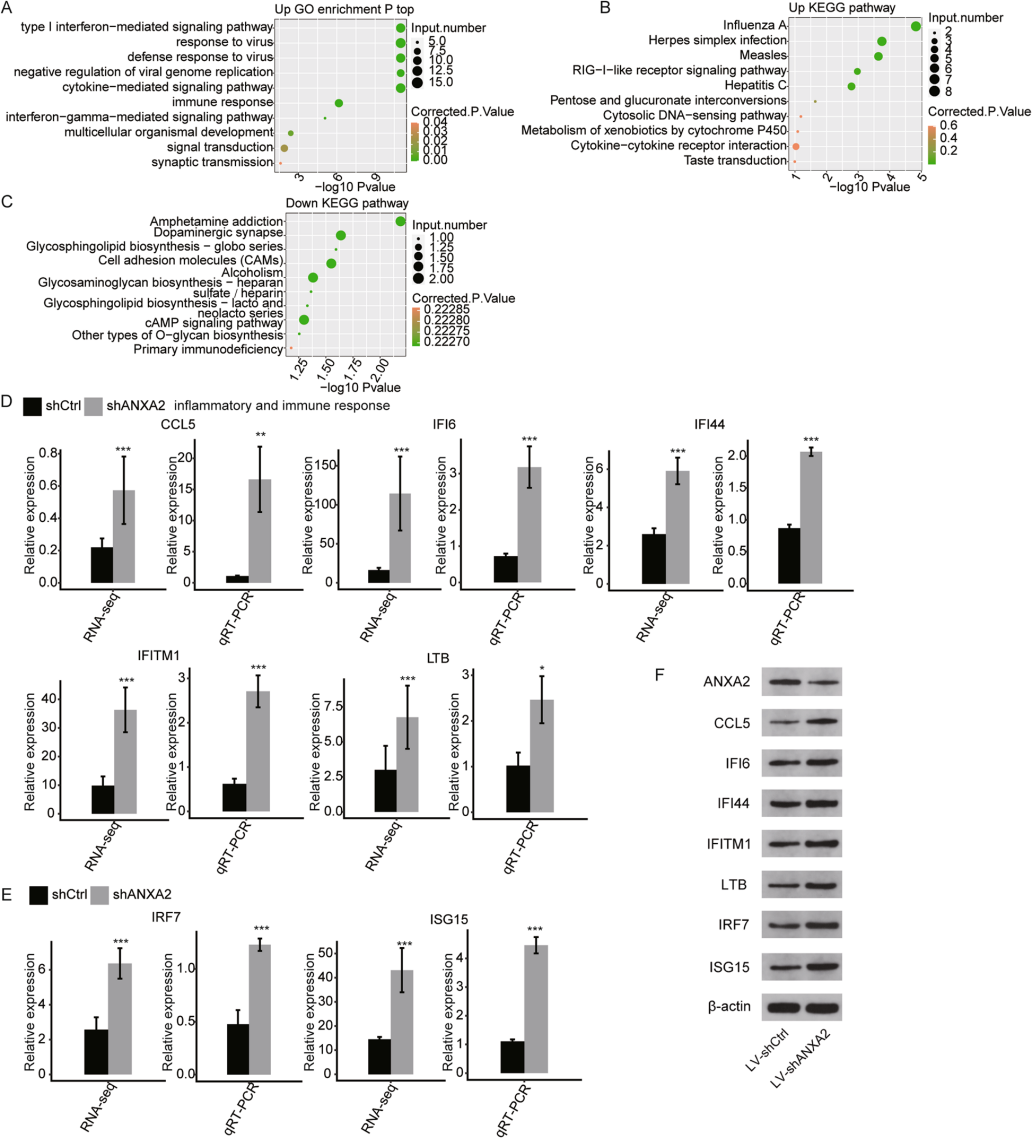
ANXA2 regulated inflammatory gene mRNA and protein expression in HK2 cells. **A** Top ten GO biological processes terms enriched by upregulated DEGs in shANXA2 cells vs shCtrl cells. **B** Top ten KEGG functional pathways enriched by upregulated DEGs in shANXA2 cells vs shCtrl cells. **C** Top ten KEGG functional pathways enriched by downregulated DEGs in shANXA2 cells vs shCtrl cells. **D** Validation of mRNA expression of CCL5, IFI6, IFI44, IFITM1,and LTB by qRT-PCR assay. **E** Validation of mRNA expression of IRF7 and ISG15 by qRT-PCR assay. **F** Representative images showing protein levels of ANXA2, CCL5, IFI6, IFI44, IFITM1, LTB, IRF7 and ISG15 in LV-shANXA2 group vs LV-shCtrl group. Results are represented as mean ± SD.(*n* = 3,* *P* < 0.05, **P < 0.01, ***P < 0.001, calculated using the student’s t-test)

In order to explore the functions of genes regulated by ANXA2 in HK2 cells, KEGG and GO pathway analyses of DEGs from shANXA2 vs. shCtrl were performed. In the GO pathway analysis of biological process, significant enrichment of upregulated genes in shANXA2 cells was found in type I interferon-mediated signaling pathway, cytokine-mediated signaling pathway, and interferon-gamma-mediated signaling pathway, with *P* value less than 0.05 (shown in Fig. 2A). The GO results of biological process in downregulated genes of shANXA2 cells was omitted since no enrichment was found. As for GO results of cellular component and and molecular function, upregulated genes in shANXA2 cells related mainly to cytoplasm, protein binding and ATP binding, while downregulated genes might enrich in membrane, cytoplasm and protein binding (shown in Fig. S1). In the KEGG pathway analysis, the upregulated genes in the ANXA2 knockdown cells were mainly associated with inflammatory pathways, including influenza A, herpes simplex infection, measles, RIG-I-like receptor signaling pathway, hepatitis C (shown in Fig. 2B). The downregulated genes were enriched in pathways such as the cell adhesion molecules and cAMP signaling pathway (shown in Fig. 2C).

In order to verify the RNA-seq results, qRT-PCR analysis was performed on seven upregulated DEGs, including *CCL5*, *IFI6*, *IFI44*, *IFITM1*, *LTB*, *IRF7*, and *ISG15*. The selected genes were enriched in cell adhesion, interferon-mediated signaling, inflammatory response pathways of the KEGG or GO analyses, and the FPKM of these genes was higher than 1 in at least one sample. The results of this experiment are presented in Fig. 2D and E. A statistically significant increase was observed in *CCL5*, *IFI6*, *IFI44*, *IFITM1*, *LTB*, *IRF7*, and *ISG15* mRNA expression in shANXA2 samples vs in shCtrl samples, in agreement with the RNA-seq analysis.

### Protein levels of inflammatory genes in stable cell lines with low ANXA2 expression and with ANXA2 overexpression

To further verify the effect of ANXA2 on the DEGs, stable cell lines with low ANXA2 expression and with ANXA2 overexpression were established. Western blot was performed to detect the corresponding protein levels of the DEGs. As shown in Fig. 2F, the protein level of ANXA2 was decreased in LV-shANXA2 group vs LV-shCtrl group. Protein levels of CCL5, IFI6, IFI44, IFITM1, LTB, IRF7, and ISG15 were increased by silencing ANXA2, which was similar to the effect of ANXA2 on gene expression of these DEGs. Additionally, overexpression of ANXA2 led to lower protein levels of these DEGs (shown in Fig. S2).

### RNA-seq analysis of ANXA2-regulated alternative splicing in HK2 cells

**Fig. 3:**
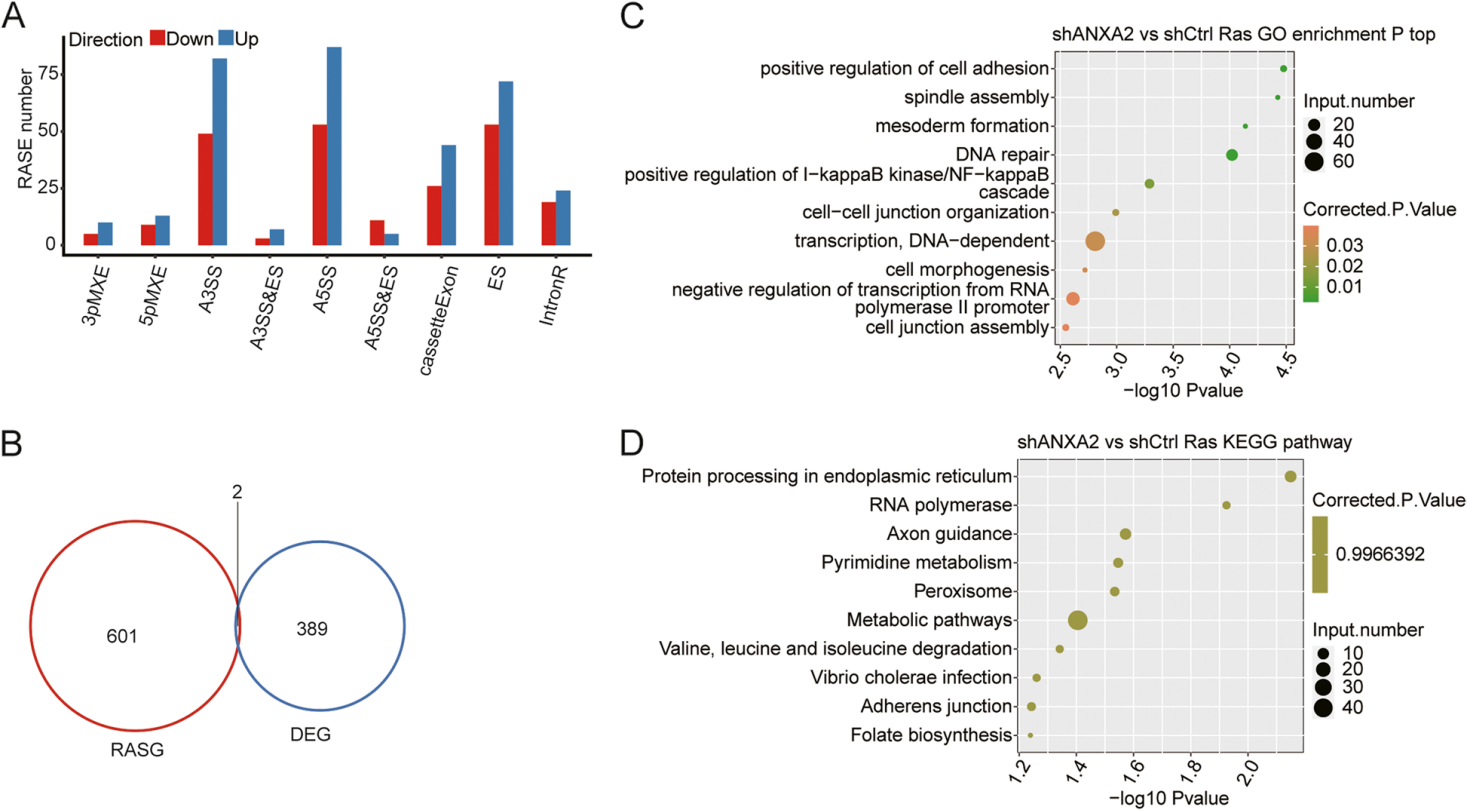
Identification and functional analysis of ANXA2-regulated splicing events. **A** Classification of regulated alternative splicing events (RASE) induced by ANXA2. **B** Analysis of the overlap between ANXA2-regulated DEGs and regulated alternative splicing genes (RASG). **C** Top ten GO biological processes terms of ANXA2-regulated alternative splicing genes. **D** Top ten KEGG functional pathways of ANXA2-regulated alternative splicing genes

The other key aim of this study was to gain insight into the role of ANXA2 on alternative splicing regulation. Therefore, we further used transcriptome sequencing data to observe the ANXA2-dependent RASEs in HK2 cells. RASEs were analyzed using ABLas software. We detected 18,491 known RASEs in the model gene that we designated in the reference genome, along with 52,724 novel RASEs, excluding intron retention (shown in Supplementary Material Table 2). When we applied a stringent cutoff of *P*<0.05 to identify high-confidence RASEs, a total of 699 RASEs were detected when comparing shANXA2 vs. shCtrl. ANXA2-regulated RASEs were summarized in Supplementary Material Table 3 and Fig. 3A, including 131 alternative 3′ splice sites (A3SS), 140 alternative 5′ splice sites (A5SS), 125 examples of exon skipping (ES), 70 cassette exons, 22 mutually exclusive 5′ untranslated regions (UTRs) (5pMXE), 15 mutually exclusive 3′ UTRs (3pMXE), 26 mutually exclusive exons (MXE), 144 examples of intron retention (IR), 16 examples of alternative 5′ splice site & exon skipping (A5SS & ES), and 10 examples of alternative 3′ splice site & exon skipping (A3SS & ES). Coupled to the transcription data, there were only 2 genes under significant transcriptional control overlapped with those under splicing control (shown in Fig. 3B). To identify the pathways in which the RASEs were mainly involved, GO and KEGG enrichment analyses were conducted. The alternative splicing genes were enriched in positive regulation of cell adhesion, positive regulation of I-κB kinase/NF-κB cascade, and cell-cell junction in GO analysis (shown in Fig. 3C). Though the KEGG analysis showed some possible enrichment, the large *P* value suggested it might not be significant enough (shown in Fig. 3D).

### ANXA2-regulated alternative splicing of key genes in inflammation signaling pathways

**Fig. 4:**
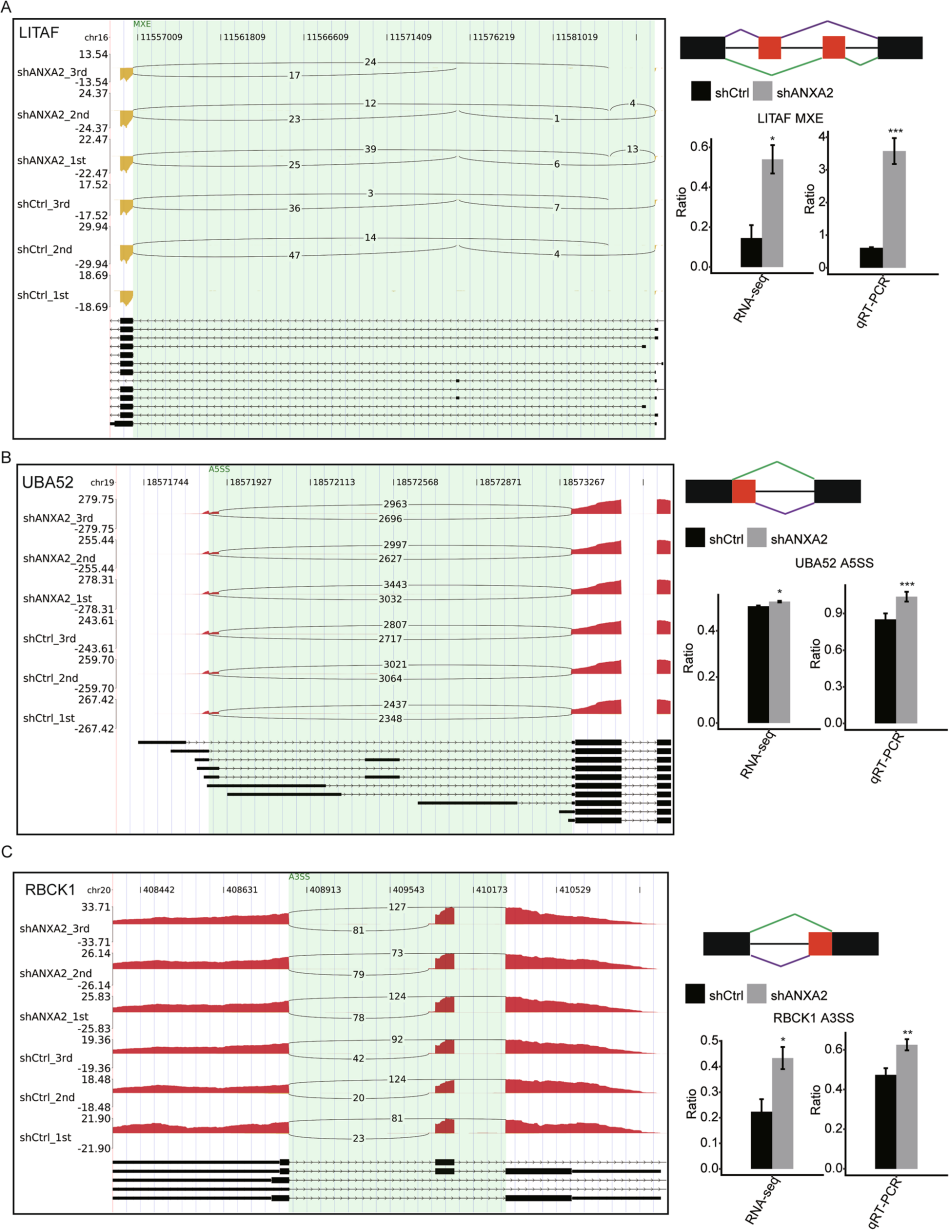
Validation of ANXA2-regulated alternative splicing events in key genes of inflammation pathways. **A** RASEs in LITAF. **B** RASEs in UBA52. **C** RASEs in RBCK1. The altered ratio of alternative splicing (AS) events in RNA-seq was calculated using the formula: AS junction reads / (AS junction reads + Model junction reads); while the altered ratio of AS events in qRT-PCR was calculated using the formula: AS transcripts level / Model transcripts level. (*n* = 3, *P < 0.05,**P < 0.01, ***P < 0.001, calculated using the student’s t-test)

To validate ANXA2-related RASEs detected by RNA-seq in HK2 cells, ten RASEs were selected for qRT-PCR analysis. Out of these ten tested events, five RASEs validated by qRT-PCR were consistent with the RNA-seq results. These five RASEs located in five relevant genes (*MAP3K3*, *UBA52*, *LITAF*, *RBCK1*, and *NOD1*). Figure 4 shows the three RASEs located in three key genes (*LITAF*, *UBA52*, and *RBCK1*) involved in inflammation pathways. These results confirmed the ANXA2-regulated RASEs identified by ABLas analysis of RNA-seq results and showed that ANXA2 was involved in alternative splicing of inflammatory genes in renal tubular epithelial cells.

## Discussion

The purpose of this study was to provide new insights into the regulation of ANXA2 on gene expression in proximal tubular cells using HK2 cells. The expression of ANXA2 in HK2 cells was silenced using shRNA. Through genome-wide transcriptional and RASE analysis of shANXA2 and shCtrl HK2 cells using RNA-seq, it was shown that ANXA2 regulated the expression and RASE of inflammatory response genes.

Transcriptional analysis identified 220 upregulated and 171 downregulated genes related to ANXA2 knockdown. DEG analysis indicated that these DEGs were enriched in multiple KEGG pathways and GO functional terms, which was consistent with the reported function of ANXA2 in signal transduction, organismal development and metastasis [23, 24]. Interestingly, ANXA2-regulated genes were also enriched in inflammatory pathways, including influenza A, herpes simplex infection, measles, hepatitis C, type I interferon-mediated signaling pathway, cytokine-mediated signaling pathway, and interferon-gamma- mediated signaling pathway. This implied that ANXA2 possibly had an important regulatory function on inflammatory response in proximal tubular cells.

During the evolution of kidney fibrosis, it was common that macrophages and T lymphocytes accumulated around the renal vasculature and throughout the interstitium of the kidney [25]. These infiltrating cells generated a milieu of pro-inflammatory molecules that exacerbate tissue injury [26]. CCL5 was reported to recruit mononuclear cells into injured tissues and some studies have shown that CCL5 dysregulation played a key role in driving the increase and activation of inflammatory macrophages in kidney disease [27]. In this study, both RNA-seq analysis and qRT-PCR showed that CCL5 was up-regulated in ANXA2 knockdown HK2 cells. This indicated that ANXA2 might regulate renal inflammation by regulating the expression of CCL5 in proximal tubular cells.

Both type I interferon and IFN-γ signaling was involved in inflammatory injury, viral infection, cell growth regulation, apoptosis, and immune responses [28, 29]. A previous study showed that a type I IFN-dependent transcript, ISG15 dysregulation, was involved in the pathogenesis of renal inflammation [30]. Furthermore, some studies have shown that IFN-*γ*-inducible protein 10 was strongly involved in the development of renal diseases [31, 32]. These findings suggested that IFN signaling might be associated with the pathogenesis of various kidney diseases and further study was desirable to discover future therapeutic strategies for kidney disease. In this study, we validated certain DEGs and discovered some elevated IFN-response genes, including *ISG15*, *IFI6*, *IFI44*, *IFITM1*, and *IRF7* in HK2 cells from the ANXA2 knockdown group compared with the control group. This indicated that ANXA2 might regulate renal inflammation by regulating the expression of IFN-response genes.

Additionally, the current study determined that ANXA2 also regulated alternative splicing of inflammatory genes, such as genes enriched in NF-κB pathways. NF-κB is one of the most important transcription factors involved in the pathophysiology of renal inflammation and fibrosis. In non-stimulated cells, NF-κB dimers are sequestered by a family of inhibitors known as IκBs (inhibitors of NF-κB). The activation of NF-κB depends on degradation of IκBs by IκB kinase (IKK), which involves signal assembling process of connecting upstream signal generation apparatuses to IKK using ubiquitin modifications. RBCK1, MAP3K3, and NOD1 were all believed to play important roles during the assembling process [33–35]. Here, it was demonstrated that ANXA2 regulated alternative splicing of these three signaling molecules, implying that ANXA2 might regulate inflammatory response partly by regulating NF-κB signaling pathways. Interestingly, a previous study verified the important role of ANXA2 in activating NF-κB signaling pathways in adipose tissue [36]. In addition, we also validated the regulatory effect of ANXA2 on RASEs in the gene *LITAF*, which was proved to be a key gene in the inflammatory response [37]. Herein, we demonstrated the regulatory effect of ANXA2 on alternative splicing of key genes in inflammatory pathways in proximal tubular cells.

## Conclusion

In this study, we successfully applied RNA-seq technology to demonstrate that ANXA2 regulated gene transcription and alternative splicing, which was consistent with its reported role as a RNA-binding protein. We showed that ANXA2 regulated transcription, alternative splicing and protein expression of genes involved in inflammatory response in HK2 cells. Our results underlined that the well-known multifunctional protein ANXA2 might play a key role in kidney inflammation. More detailed information and the underlying molecular mechanism of ANXA2 on kidney inflammation need to be further elucidated. This might contribute to precise understanding of signaling networks directing kidney inflammation, and potentially ANXA2-targeted therapies.

## Methods

### Plasmid construction

ANXA2-specific short hairpin RNA (shRNA) was designed using the free software OptiRNA. The silence sequence was TGAGGGTGACGTTAGCATTAC and was synthesized by TianyiHuiyuan Biotechnology Co., LTD (Beijing, China). The vector pGFP-B-RS (OriGene, Rockville, MD, USA) was digested using HindIII (NEB, Beijing, China) and BamHI (NEB, Beijing, China) and purified with a Qiagen column kit (Qiagen, Dusseldorf, Germany). Sense and antisense strands were annealed to the shRNA and inserted to the processed vector by T4 DNA Ligase (NEB, Beijing, China). *Escherichia coli* (DH5α, ECOS, fye607s) containing the plasmids were plated onto LB plates and incubated overnight at 37 °C. Colonies were screened by colony PCR (30 cycles) with universal primers. The interference sequence of shRNA was verified by Sanger sequencing.

### Cell culture and transfections

Human proximal renal tubular epithelial (HK2) cells were obtained from Procell Life Science & Technology Co., Ltd. (Wuhan, Hubei, China). HK2 cells were cultured at 37 °C with 5% CO_2_ in Dulbecco’s modified Eagle’s medium contaning 100 U/mL penicillin, 100 μg/mL streptomycin and 10% fetal bovine serum. Lipofectamine 2000 (Invitrogen, Carlsbad, CA, USA) was utilized to transfect plasmids into HK2 cells. Transfected HK2 cells were cultured for 48 h before quantitative real-time PCR (qRT-PCR) analysis. ANXA2 primers: Forward, ATGTTCCCAAGTGGATCAGC and reverse, ACAGGGGCTTGTTCTGAATG. The primers were synthesized by TianyiHuiyuan Biotechnology Co., LTD (Beijing, China). Glyceraldehyde 3-phosphate dehydrogenase (*GAPDH*) was adopted as internal standard and gene expression was evaluated by the 2-ΔΔCq method.

### Establishment of stable transfectants

ANXA2-specific shRNA sequence was cloned into the lentiviral vector FV023 (Fubio, Suzhou, China) to construct stable cell lines with low ANXA2 expression(LV-shANXA2 group), while the control group was loaded with a scramble RNA sequence (LV-shCtrl group). ANXA2 sequence were cloned into lentiviral vector FV026 (Fubio,Suzhou,China) to generate stable cell lines with ANXA2 overexpression (LV-OE-ANXA2 group), with empty FV026 as control (LV-OE-Ctrl). HK2 cells at 70–80% confluence were infected by respective lentiviruses with the multiplicity of infection (MOI) of 100. After 48 h post infection, positive HK2 cells were selected with 2 μg/mL puromycin (Sigma, USA) for 14 days. Western blot was then used to detect ANXA2 protein levels of each group.

### RNA extraction and sequencing

Total RNA was extracted using TRIzol (Invitrogen, Carlsbad, CA, USA), followed by RQ1 DNase (Promega, Madison, WI, USA) treatment to eliminate DNA. Absorbance at 260 nm/280 nm (A260/A280), along with 1.5% agarose gel electrophoresis were performed to standardize the quality and integrity of RNA samples. For RNA-seq library preparation, oligo(dT)-conjugated magnetic beads (Invitrogen, Carlsbad, CA, USA) were adopted to purify the samples and 1 μg of each RNA sample underwent reverse transcription into cDNA using the PrimeScript™ RT Reagent Kit (Takara, Dalian, China). Libraries for high-throughput sequencing were prepared according to the manufacturer’s instructions and applied to the Illumina HiSeq X Ten system for 150-nt paired-end sequencing.

### Bioinformatics analysis

Raw reads were filtered to remove the adaptors, PolyN reads, short reads less than 16 nt, and low-quality bases, using FASTX-Toolkit (version 0.0.13). Clean reads were then aligned to the GRCh38 genome using TopHat2 software [38] with four mismatches. Uniquely mapped reads were used to calculate the reads number and FPKM value (fragments per kilobase per million mapped reads). Raw read counts were used as input in edgeR to discriminate the differentially expressed genes (DEGs) [39]. Genes with lower FPKM than 0.1 were not considered for further analysis, while genes with considerable log2 fold change (>1 or <-1) and *P* value (*P <* 0.05) were identified as DEGs. Gene Ontology (GO, http://www.geneontology.org) and Kyoto Encyclopedia of Genes and Genomes (KEGG, http://www.kegg.jp) analyses were performed to explore the potential function of DEGs. Bioinformatics resources were obtained from the database for annotation, visualization, and integrated discovery (DAVID) bioinformatics resources (https://david.ncifcrf.gov/). ABLas pipeline was used to calculate the regulated alternative splicing events (RASEs) [40]. A *P*-value of < 0.05 and a RASE ratio of > 0.2 was set as the threshold for RASE detection.

### Validation of DEGs and RASEs

To validate the DEGs and RASEs, expression of several genes were evaluated by qRT-PCR on a Bio-Rad S1000 thermal cycler with Bestar SYBR Green RT-PCR Master Mix (DBI Bioscience, Shanghai, China). Supplementary Material Table 4 presented the primers for DEGs and Supplementary Material Table 5 for RASEs. The PCR procedure was denaturing at 95 °C for 10 min, 40 cycles of denaturing at 95 °C for 15 s, annealing and extension at 60 °C for 1 min. For each sample, three experimental replicates were performed.

### Western blot

Protein samples were extracted from cultured HK2 cells with RIPA lysis buffer (Beyotime, China) with the presence of 1 mM phenylmethane-sulfonyl fluoride (PMSF). Equal amount of each sample was electrophoresed by SDS-PAGE gels and then transferred to polyvinylidene difluoride membranes (PVDF, Millipore, USA). The membranes were incubated overnight separately with specific rabbit primary antibodies after 2 h of blocking. Anti-rabbit secondary antibody conjugated to horseradish peroxidase (Boster, BA1054, Wuhan, China) was incubated with the membranes for 1 h. Primary antibodies anti-ANXA2(AF 5420), anti-CCL5 (DF5151), anti-IFI6 (DF10115), anti-IFITM1 (DF 2513), anti-LTB (DF 3243), anti-IRF7 (DF7503), and ISG15 (DF6316) were from Affinity Biosciences (Cincinnati, OH, USA). Other primary antibodies included anti-IFI44 (Proteintech, 27,233–1-AP,Wuhan, China) and anti-actin (Boster, BM3873, Wuhan, China).

### Statistical analysis

All values were presented as mean ± SD. For comparison, the significance of differences between means was determined using the Student’s *t-*test. A *P* value of *<* 0.05 was regarded as statistically significant.

## Supplementary Information

**Additional file 1: Table 1.** Summary of RNA-seq reads used in the analysis.

**Additional file 2: Table 2.** Known and novel splicing events detected using ABLas. ^a^Numbers in this line indicate the unique RASEs identified from all four samples. Since many RASEs were detected in more than one sample, the numbers in this line are less than the sum of the four individual numbers. ^b^ Indicates the sum of all types of RASEs detected in each sample or all samples. ^c^ Indicates the known spliced junctions detected in each sample or all samples. RASEs, regulated alternative splicing events.

**Additional file 3: Table 3.** Summary of ANXA2-regulated alternative splicing events.

**Additional file 4: Table 4.** Primer sequences used in qRT-PCR experiments for DEG validation.

**Additional file 5: Table 5.** Primer sequences used in qRT-PCR experiments for RASE validation.

**Additional file 6: Fig. S1.** GO analysis of cellular component and molecular function of DEGs in shANXA2 vs shCtrl cells. (A) GO cellular component terms enriched by upregulated DEGs in shANXA2 cells vs shCtrl cells.(B) GO cellular component terms enriched by downregulated DEGs in shANXA2 cells vs shCtrl cells.(C) GO molecular function terms enriched by upregulated DEGs in shANXA2 cells vs shCtrl cells.(D) GO molecular function terms enriched by downregulated DEGs in shANXA2 cells vs shCtrl cells.

**Additional file 7: Fig. S2.** Representative images showing protein levels of ANXA2, CCL5, IFI6, IFI44, IFITM1, LTB, IRF7 and ISG15 in LV-OE-ANXA2 group vs LV-OE-Ctrl group.

**Additional file 8: Fig. S3.** Original images showing protein bands of ANXA2, CCL5, IFI6, IFI44, IFITM1, LTB, IRF7 and ISG15 in LV-shANXA2 group vs LV-shCtrl group, and in LV-OE-ANXA2 group vs LV-OE-Ctrl group.

## Data Availability

The datasets generated and/or analysed during the current study are available in the GEO database (https://www.ncbi.nlm.nih.gov/geo/query/acc.cgi?acc=GSE159360, accession number GSE159360).

## Abbreviations

ANXA2: Annexin A2
CKD: Chronic kidney disease
DEGs: Differentially expressed genes
RASEs: Regulated alternative splicing events
NF-κB: Nuclear factor κB
IFN-γ: Interferon-γ
TNF-α: Tumor necrosis factor-α
RNA-seq: RNA-sequencing
shRNA: Short hairpin RNA
GO: Gene Ontology
KEGG: Kyoto Encyclopedia of Genes and Genomes
DAVID: Database for annotation, visualization, and integrated discovery
FPKM: Fragments per kilobase of exon model per million fragments mapped
FDR: False discovery rate
IκBs: Inhibitors of NF-κB
IKK: IκB kinase
RASG: Regulated alternative splicing genes
