# Supplementary material for: Annexin A2 (ANXA2) regulates the transcription and alternative splicing of inflammatory genes in renal tubular epithelial cells
Source: BMC Genomics. 2022 Jul 29;23:544. doi: 10.1186/s12864-022-08748-6 (PMC9336024; doi:10.1186/s12864-022-08748-6)
Supplement: Supplementary file 1 — Additional file 1: Table 1. Summary of RNA-seq reads used in the analysis. [file 12864_2022_8748_MOESM1_ESM.docx]

Table 1. Summary of RNA-seq reads used in the analysis

| Sample | Raw reads | Clean reads | Paired‑end reads | Total mapped | Total uniquely mapped |
| --- | --- | --- | --- | --- | --- |
| shANXA2_1st | 86319172 | 82866385 | 79770956 | 77216775(96.8%) | 71221598(92.24%) |
| shANXA2_2nd | 79643310 | 76812077 | 74214862 | 72069214(97.11%) | 68825173(95.5%) |
| shANXA2_3rd | 77169208 | 72255641 | 68688744 | 66036992(96.14%) | 59234131(89.7%) |
| shCtrl_1st | 64925588 | 60161283 | 57323402 | 55142943(96.2%) | 50130858(90.91%) |
| shCtrl_2nd | 77617396 | 74419324 | 71617666 | 69205023(96.63%) | 66440982(96.01%) |
| shCtrl_3rd | 75330052 | 72371395 | 69680998 | 67427901(96.77%) | 64042184(94.98%) |
| mean±SD | 76834121±6966475 | 75182702±7963204 | 70216105±7457755 | 67849808±7379821 | 63315821±7663575 |
