# Supplementary material for: Annexin A2 (ANXA2) regulates the transcription and alternative splicing of inflammatory genes in renal tubular epithelial cells
Source: BMC Genomics. 2022 Jul 29;23:544. doi: 10.1186/s12864-022-08748-6 (PMC9336024; doi:10.1186/s12864-022-08748-6)
Supplement: Supplementary file 2 — Additional file 2: Table 2. Known and novel splicing events detected using ABLas. aNumbers in this line indicate the unique RASEs identified from all four samples. Since many RASEs were detected in more than one sample, the numbers in this line are less than the sum of the four individual numbers. b Indicates the sum of all types of RASEs detected in each sample or all samples. c Indicates the known spliced junctions detected in each sample or all samples. RASEs, regulated alternative splicing events. [file 12864_2022_8748_MOESM2_ESM.docx]

Table 2. Known and novel splicing events detected using ABLas

| Known splicing events | | | | | | | | | | | | |
| --- | --- | --- | --- | --- | --- | --- | --- | --- | --- | --- | --- | --- |
|  | **3pMXE** | 5**pMXE** | **A3SS** | **A3SS**  **&ES** | **A5SS** | **A5SS**  **&ES** | **ES** | **IntronR** | **MXE** | **CassetteExon** | **Total^b^** | **Detected junction^c^** |
| shANXA2  _1st | 320 | 567 | 2847 | 306 | 3254 | 425 | 2173 | 1140 | 255 | 1536 | 12823 | 140800 |
| shANXA2  _2nd | 317 | 557 | 2835 | 307 | 3172 | 419 | 2149 | 1160 | 257 | 1553 | 12726 | 142112 |
| shANXA2  _3rd | 259 | 417 | 2191 | 243 | 2423 | 331 | 1692 | 1062 | 178 | 1173 | 9969 | 125308 |
| shCtrl  _1st | 171 | 322 | 1617 | 178 | 1791 | 225 | 1271 | 1031 | 126 | 828 | 7560 | 113549 |
| shCtrl  _2nd | 286 | 463 | 2594 | 267 | 2762 | 371 | 1957 | 1143 | 238 | 1369 | 11450 | 135054 |
| shCtrl  _3rd | 337 | 562 | 2989 | 320 | 3318 | 447 | 2292 | 1193 | 299 | 1634 | 13391 | 144783 |
| Total^a^ | 484 | 848 | 4107 | 435 | 4677 | 601 | 3021 | 1645 | 431 | 2242 | 18491 | 161682 |
| Novel splicing events | | | | | | | | | | | | |
| sample | **3pMXE** | 5**pMXE** | **A3SS** | **A3SS**  **&ES** | **A5SS** | **A5SS**  **&ES** | **ES** | **IntronR** | **MXE** | **CassetteExon** | **Total^b^** | **Detected junction^c^** |
| shANXA2  _1st | 728 | 1571 | 4654 | 518 | 4728 | 536 | 1739 | 3005 | 246 | 788 | 18513 | 58481 |
| shANXA2  _2nd | 718 | 1375 | 4250 | 439 | 4365 | 479 | 1528 | 3102 | 225 | 734 | 17215 | 58137 |
| shANXA2  _3rd | 587 | 1156 | 3886 | 423 | 3862 | 447 | 1569 | 2627 | 152 | 612 | 15321 | 34713 |
| shCtrl  _1st | 351 | 733 | 2684 | 302 | 2862 | 370 | 1226 | 2482 | 114 | 366 | 11490 | 20604 |
| shCtrl  _2nd | 573 | 1197 | 4019 | 424 | 4130 | 466 | 1479 | 3080 | 211 | 626 | 16205 | 44717 |
| shCtrl  _3rd | 670 | 1393 | 4147 | 456 | 4157 | 497 | 1559 | 3154 | 250 | 770 | 17053 | 66856 |
| Total^a^ | 2024 | 3957 | 13510 | 1682 | 13607 | 1647 | 4806 | 8612 | 666 | 2213 | 52724 | 154634 |
