# Supplementary material for: Annexin A2 (ANXA2) regulates the transcription and alternative splicing of inflammatory genes in renal tubular epithelial cells
Source: BMC Genomics. 2022 Jul 29;23:544. doi: 10.1186/s12864-022-08748-6 (PMC9336024; doi:10.1186/s12864-022-08748-6)
Supplement: Supplementary file 3 — Additional file 3: Table 3. Summary of ANXA2-regulated alternative splicing events. [file 12864_2022_8748_MOESM3_ESM.docx]

Table 3. Summary of ANXA2-regulated alternative splicing events

| sample | type | **3pMXE** | 5**pMXE** | **A3SS** | **A3SS &ES** | **A5SS** | **A5SS**  **&ES** | **ES** | **IntronR** | **MXE** | **CassetteExon** | **Total** |
| --- | --- | --- | --- | --- | --- | --- | --- | --- | --- | --- | --- | --- |
| shANXA2_vs_shCtrl | up | 10 | 13 | 82 | 7 | 87 | 5 | 72 | 70 | 15 | 44 | 405 |
| shANXA2_vs_shCtrl | down | 5 | 9 | 49 | 3 | 53 | 11 | 53 | 74 | 11 | 26 | 294 |
