# Supplementary material for: Annexin A2 (ANXA2) regulates the transcription and alternative splicing of inflammatory genes in renal tubular epithelial cells
Source: BMC Genomics. 2022 Jul 29;23:544. doi: 10.1186/s12864-022-08748-6 (PMC9336024; doi:10.1186/s12864-022-08748-6)
Supplement: Supplementary file 4 — Additional file 4: Table 4. Primer sequences used in qRT-PCR experiments for DEG validation. [file 12864_2022_8748_MOESM4_ESM.docx]

Table 4. Primer sequences used in qRT-PCR experiments for DEG validation

| DEGs | Forward | Reverse |
| --- | --- | --- |
| CCL5 | GCCCACATCAAGGAGTATTT | ACTTGCCACTGGTGTAGAAAT |
| ISG15 | GCTCCATGTCGGTGTCAGA | AGAGGTTCGTCGCATTTGT |
| IFI6 | TATTGTCCAGGCTAGAGTGC | AATCCTACTTGGGAGGTTGA |
| IFI44 | CTTTCTGACATCTCGGTGGT | GCAGCCCATAGCATTCGT |
| IFITM1 | CCCCAAAGCCAGAAGATG | TTGAACAGGGACCAGACG |
| IRF7 | CCTAGCAGCAGGGGAGGT | GCAGCATCACGAAGCGAC |
| LTB | GGCCAGGAGACAAGGGTA | TCGCACCACGCACTCA |
