# Supplementary material for: Annexin A2 (ANXA2) regulates the transcription and alternative splicing of inflammatory genes in renal tubular epithelial cells
Source: BMC Genomics. 2022 Jul 29;23:544. doi: 10.1186/s12864-022-08748-6 (PMC9336024; doi:10.1186/s12864-022-08748-6)
Supplement: Supplementary file 5 — Additional file 5: Table 5. Primer sequences used in qRT-PCR experiments for RASE validation. [file 12864_2022_8748_MOESM5_ESM.docx]

Table 5. Primer sequences used in qRT‑PCR experiments for RASE validation

| RASE | Model forward | AS forward | Model/AS reverse |
| --- | --- | --- | --- |
| NOD1 | CGCATCTTAAACTGGGAAAA | TCGGAGCCAGACTGGGAAAA | CCAACCTCAGAGATTGATTT |
| MAP3K3 | CGTTCCTGAGGTGACACTCA | GGCTGCGGAGGTGACACTCA | CCATGGTGGCGATG |
| LITAF | CGGGACACAGGTAAAATGTC | GGTCTATCAGGTAAAATGTC | TAGTTGGCCCAGGCAT |
| UBA52 | GGCCGAGCTG ACGCAAACAT | GGTCGTGCGGACGCAAACAT | CACTGGGCTCGACCTCAA |
| RBCK1 | CATGAGGAATCTTTCTTGGT | TTTTCAGGAGCTTTCTTGGT | GTGATGCTTCCCGACTGC |
