# Supplementary figures and images for: Annexin A2 (ANXA2) regulates the transcription and alternative splicing of inflammatory genes in renal tubular epithelial cells
Source: BMC Genomics. 2022 Jul 29;23:544. doi: 10.1186/s12864-022-08748-6 (PMC9336024; doi:10.1186/s12864-022-08748-6)

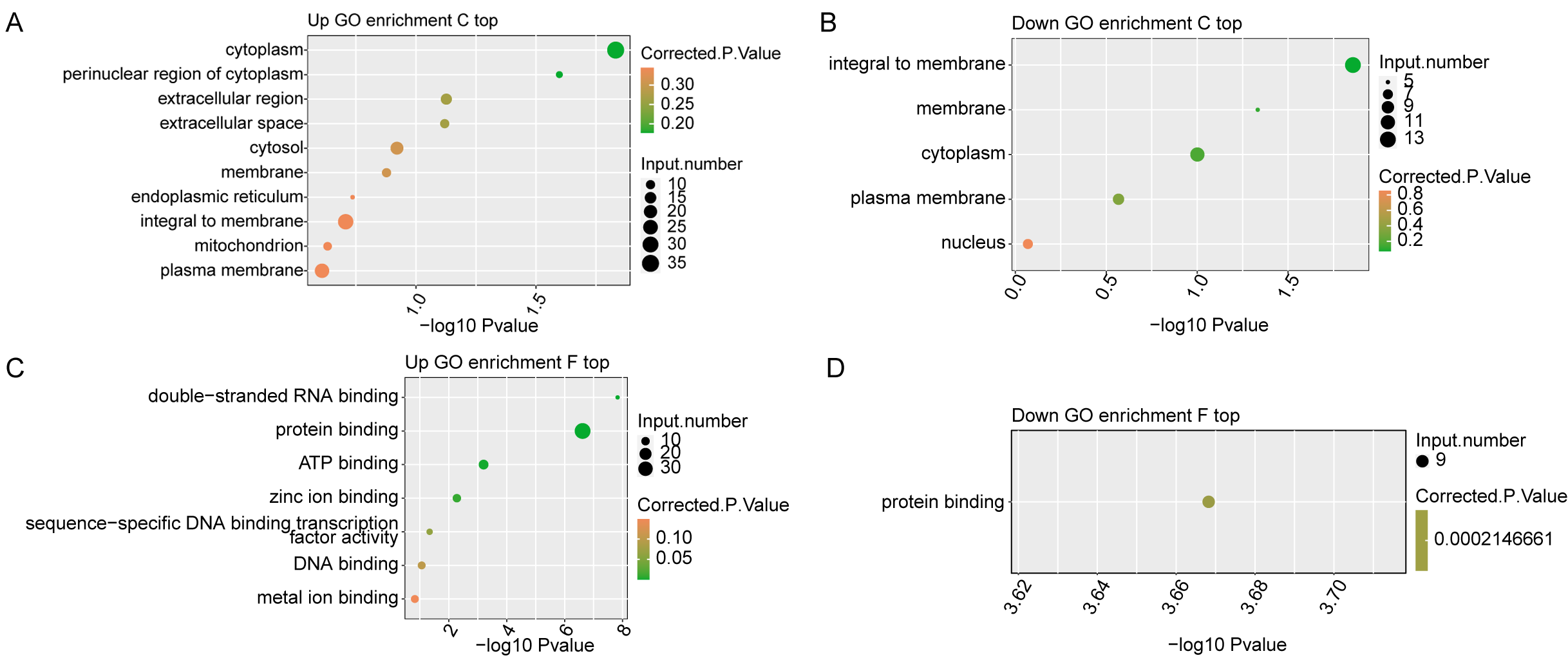

Supplement: Supplementary file 6 — Additional file 6: Fig. S1. GO analysis of cellular component and molecular function of DEGs in shANXA2 vs shCtrl cells. (A) GO cellular component terms enriched by upregulated DEGs in shANXA2 cells vs shCtrl cells.(B) GO cellular component terms enriched by downregulated DEGs in shANXA2 cells vs shCtrl cells.(C) GO molecular function terms enriched by upregulated DEGs in shANXA2 cells vs shCtrl cells.(D) GO molecular function terms enriched by downregulated DEGs in shANXA2 cells vs shCtrl cells. [file 12864_2022_8748_MOESM6_ESM.tif]

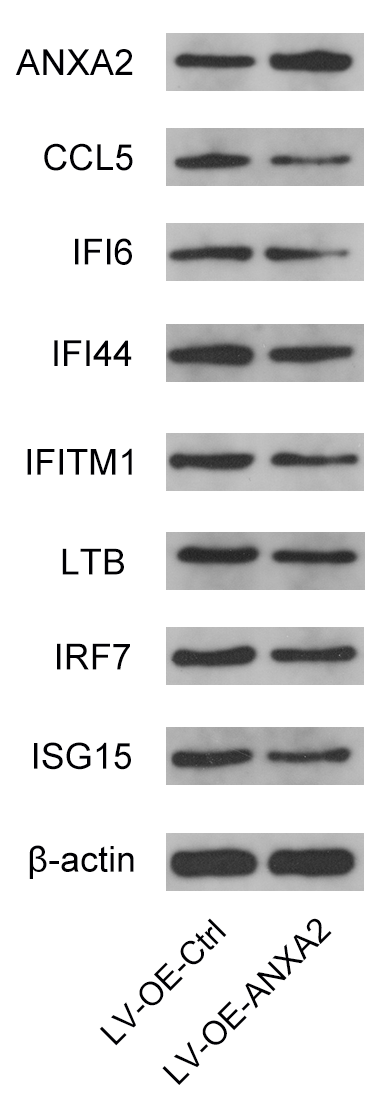

Supplement: Supplementary file 7 — Additional file 7: Fig. S2. Representative images showing protein levels of ANXA2, CCL5, IFI6, IFI44, IFITM1, LTB, IRF7 and ISG15 in LV-OE-ANXA2 group vs LV-OE-Ctrl group. [file 12864_2022_8748_MOESM7_ESM.tif]

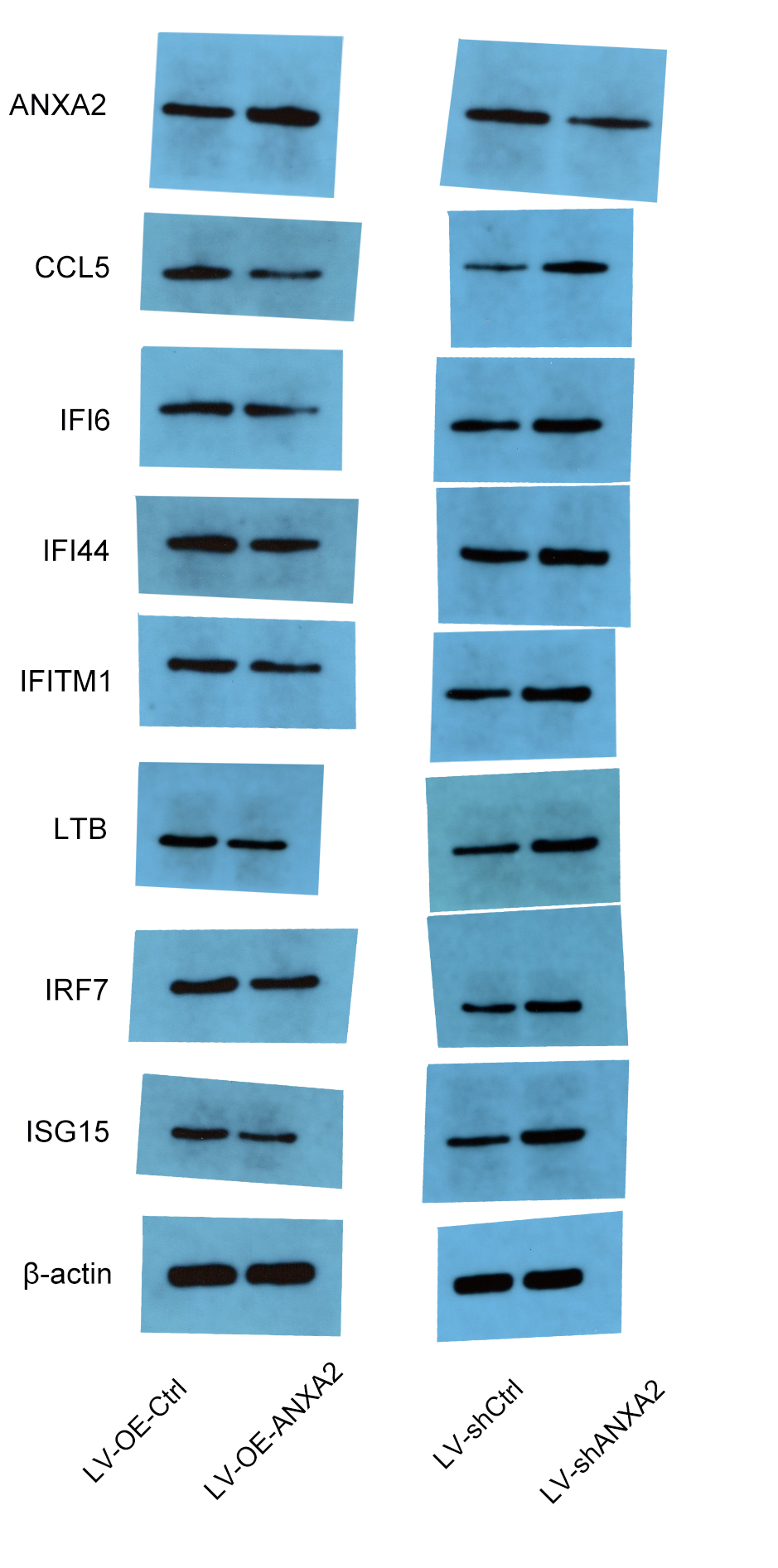

Supplement: Supplementary file 8 — Additional file 8: Fig. S3. Original images showing protein bands of ANXA2, CCL5, IFI6, IFI44, IFITM1, LTB, IRF7 and ISG15 in LV-shANXA2 group vs LV-shCtrl group, and in LV-OE-ANXA2 group vs LV-OE-Ctrl group. [file 12864_2022_8748_MOESM8_ESM.jpg]
